# Heath related quality of life and associated factors among diabetes patients in sub-Saharan countries: a systemic review and meta-analysis

**DOI:** 10.1186/s12955-020-01655-y

**Published:** 2021-01-25

**Authors:** Biruk Shalmeno Tusa, Adisu Birhanu Weldesenbet, Assefa Tola Gemada, Bedasa Taye Merga, Lemma Demissie Regassa

**Affiliations:** 1 Department of Epidemiology and Biostatistics, Collage of Health and Medical Sciences, Haramaya University, Haramaya, Ethiopia; 2 Department of Public Health and Health Policy, School of Public Health, Collage of Health and Medical Sciences, Haramaya University, Haramaya, Ethiopia

**Keywords:** Diabetes mellitus, Heath related quality of life, Sub-Saharan, Systemic review

## Abstract

**Background:**

Various primary studies have been conducted in sub-Saharan countries on the level of health related quality of life (HRQoL) and their associated factors among diabetic patients. However, the result of these studies lacks consistency. Therefore, this systematic review and meta-analysis estimates the pooled level of HRQoL and their associated factors among diabetic patients in sub-Saharan countries.

**Methods:**

Electronic databases predominantly PubMed were searched. Databases, such as Google and Google scholar, were searched for gray literature. A funnel plot and Egger’s regression test were used to see publication bias. Heterogeneity of the studies was checked by Forest plot and I-squared statistic. Both inverse-variance fixed-effect and DerSimonian and Laird random-effects methods were applied to estimate the pooled level of HRQoL (for both WHO-QoL-BREF and SF-36) and the effect size of associated factors.

**Result:**

From a total 776 retrieved studies, 16 studies were included for systematic review and meta-analysis. The pooled mean score of physical health, psychological, social relation and environmental health domain of WHO-QoL-BREF were 43.12, 47.40, 46.60 and 45.59 respectively. Age had a significant association (pooled β = − 0.47), (pooled β = − 0.24), (pooled β = − 0.32) and (pooled β = − 0.03) with physical health, psychological health, social relation and environmental health domains respectively. Being rural residence (pooled β = − 0.32) was inversely associated with environmental health domain of WHO-QoL-BREF. Increased fasting blood sugar had a significant association (pooled β = − 0.08, 95% CI − 0.11, − 0.05), (pooled β = − 0.07) and (pooled β = − 0.004) with physical health, psychological health and environmental health domains respectively. Having Co-morbidity (pooled β = − 6.25) and diabetes related complication (pooled β = − 5.65) were contrarily related to physical health domain of WHO-QoL-BREF.

**Conclusion:**

The pooled mean of physical and environmental domains of HRQOL scores was the least compared to the psychological and social domains. Being Old age and rural residence, increased fasting blood sugar, having co-morbidity and diabetic related complications were contrarily related to level of HRQoL. Therefore, we recommend that early detection and treatment of diabetes related complication and comorbidity and control of fasting blood sugar. While doing that due attention should be given for old and rural dwellers.

## Introduction

Diabetes mellitus (DM) is chronic disease happened due to rising blood sugar level because of the body cannot produce at all or secrets insufficient insulin hormone or not use it effectively. Hence, the absence of insulin or the cell is not sensitive to consume insulin leads to raise blood sugar level, which is the hallmark of diabetes [1].

One of the chief global public health problems now days is diabetes, particularly the burden is higher in low income countries, due to population growth, consumption of unhealthy diets, obesity, and sedentary lifestyles [2, 3]. Globally 463 million people (20–79 years of age) suffered from DM and the number is expected to rise to 700 million by 2045. An estimated 19 million adults live in Africa and this figure is estimated to increase to 47 million by 2045 [4].

Diabetes mellitus is a chronic disease, which is cause for both micro-vascular (nephropathy, retinopathy and neuropathy) and macro-vascular (stroke, coronary artery disease and diabetes foot ulcer) with co-morbidities lead to significant diminish in patient’s quality of life (QoL) as well as socio-economic consequence [5]. The world health organization defines quality of life as individuals’ perception of their position in life in which they live and in relation to their goals expectation, standard and concern [6].

In chronic diabetes patients, a complete cure cannot be attained rather clinical measures have provided for a good estimate of disease control with the ultimate goal of enhancing patient’s QOL [7]. Knowing the predictors and recognizing risk factors of QOL is essential and these factors may then be targeted for prevention [8].

Recently, many studies have been conducted in sub-Saharan countries on the level of HRQoL and their associated factors among diabetic patients [6, 9–23]. However the result of these studies lacks consistent with the level of HRQoL and even the factors that have significant association with HRQoL among diabetic patients varies across the studies. Moreover, there is no a study that shows the regional pooled level of HRQoL and contributing factors as well. Therefore, this systemic review and meta-analysis on the level of HRQoL and associated factors in sub-Saharan countries are filling these gaps and will generate new attention on the significant contributing factors to maintain the optimum level of HRQoL among diabetic patients in sub-Saharan countries.

## Methods and materials

### The protocol and registration

The results of this systematic review and meta-analysis were reported based on the Preferred Reporting Items for Systematic Review and Meta-Analysis statement (PRISMA) guideline [24]. We tracked the flowchart from the PRISMA guideline recommendation to show the selection process from initially identified records to finally included studies. The protocol for this review was registered on the International Prospective Register of Systematic Reviews (PROSPERO) registration number CRD42020165842.

### Searching strategy

**Fig. 1:**
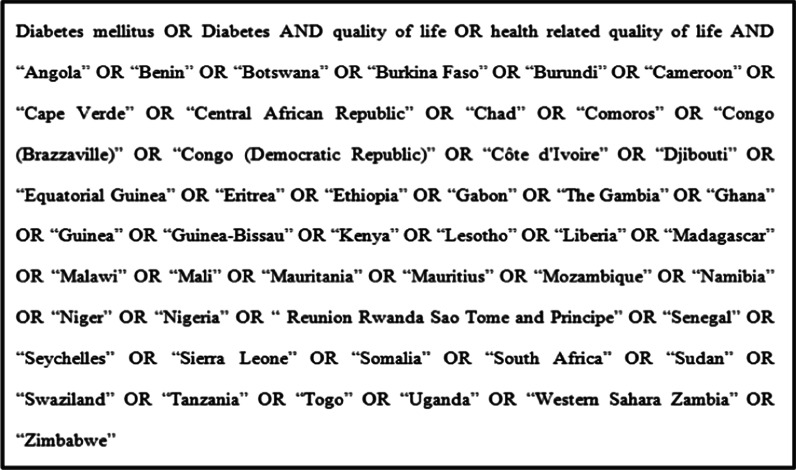
Terms used in PubMed search

Electronic databases predominantly PubMed was searched. Databases, such as Google and Google scholar, were searched for gray literature. Moreover, we emailed for nine authors to request extra information lost from their papers. However, two authors [11, 21] replied to the email request. The search was restricted to papers published in between January 1, 2000 to April 1, 2020 in sub-Saharan Africa and published in English. The core search terms and phrases were “health related quality of life”, “quality of life”, “Diabetes mellitus”,”Diabetes” and sub-Saharan countries (Fig. 1).

### Inclusion and exclusion criteria

Cross-sectional, case–control, and cohort studies that were conducted among diabetes (both type 1 and type2) patients and age greater than 18 years were included. Those studies had reported the level of HRQoL and/or at least one associated factors of level of in English language were considered. Citations without abstract and/ or full-text, anonymous reports, editorials, systematic reviews and meta-analyses and qualitative studies were excluded from the analysis.

### Study selection

Retrieved studies were exported to reference manager software, Endnote version 7 to remove duplicate studies. Initially, two independent reviewers screened the title and abstract, then full-text as per inclusion criteria. Two independent authors (AB & AT)) conducted the abstract and full-text review. The disagreement between two reviews was handled through a discussion. However, in the case of further disagreement, other authors (BS) made the final decisions.

### Quality assessment

Two independent authors (BT& LD) appraised the quality of studies. The Joanna Briggs Institute (JBI) quality appraisal checklist was used [25]. Studies were considered low risk when it scored 50% and above of the quality assessment indicators.

### Data extraction

Two independent reviewers extracted data using a structured data extraction form. Whenever variations of extracting data observed, the phase was repeated. If discrepancies between data extractors continued, third reviewer was involved. The name of the first author and year of publication, study design, sample size, objective of the study, scale, statistical model and effect size were collected.

### Statistical analysis

The Stata software 14.0 was be used to analyze the extracted data. Publication bias was checked by funnel plot and more objectively through Egger’s regression test [26]. Heterogeneity of studies was observed using forest plot and quantified using the I-squared statistic, in which 25, 50, and 75% represented low, moderate and high heterogeneity respectively [27, 28]. Both inverse-variance fixed-effect and DerSimonian and Laird random-effects methods were applied to estimate the pooled level of HRQoL and the effect size of associated factors[29].

## Result

### Study characteristics

**Fig. 2:**
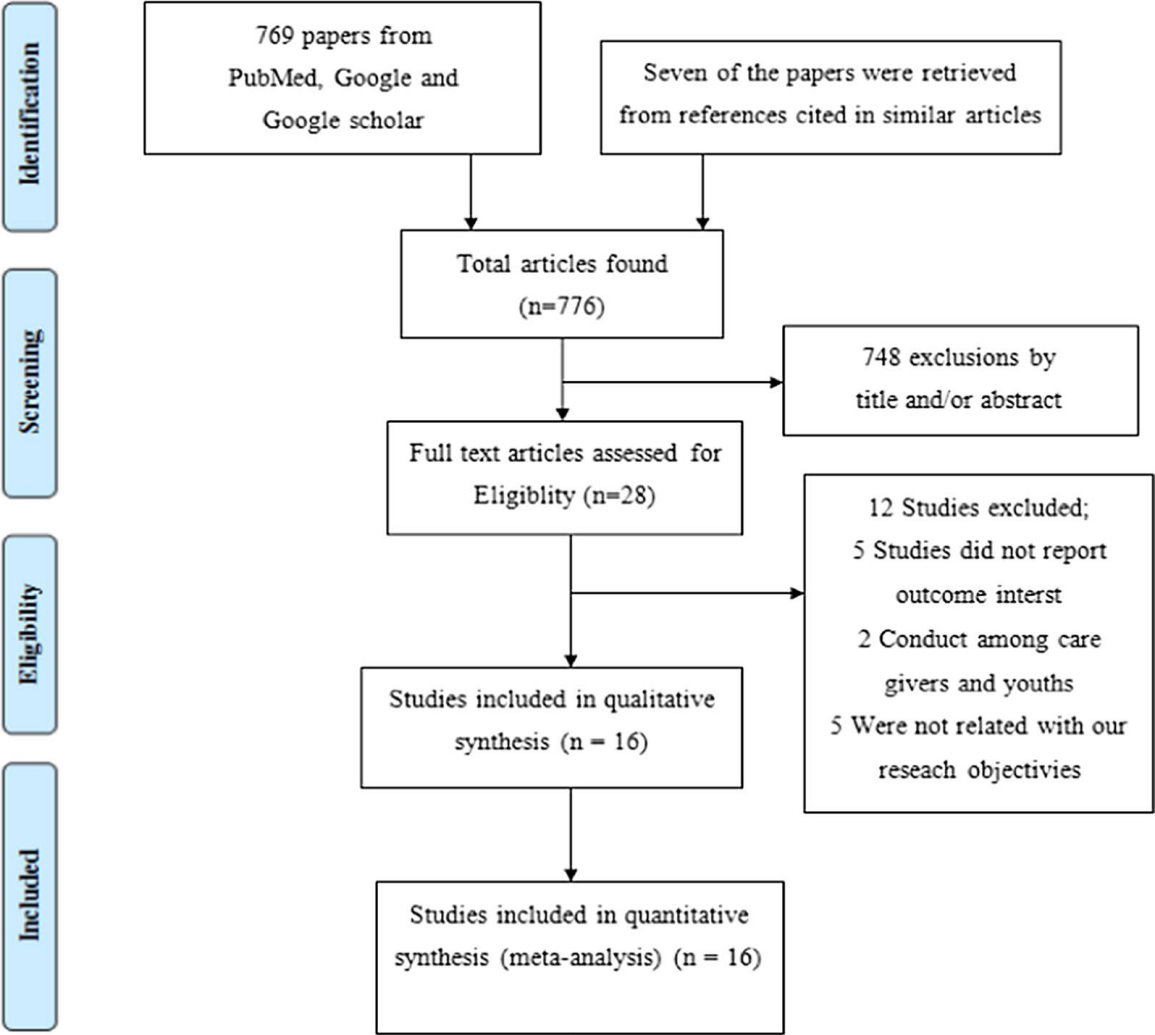
PRISMA flow diagram for article selection and screening

The search retrieved a total of 776 studies. 748 articles were excluded based on the title and abstract screen. Full-text reviews were conducted on the remaining 28 studies. Finally, we included 16 studies for qualitative synthesis and quantitative synthesis, respectively (Fig. 2).

Table 1 describes the characteristics of the studies included in this review. Five studies were found in Ethiopia [10, 14, 18, 20, 22, 30], four in Nigeria [11, 13, 15], two in South Africa [12, 23], one in Botswana [21], Ghana and Nigeria [9], Mauritius [16], Swaziland [17] and Uganda [19]. All of the studies were done by cross-sectional study design. Regarding year of publication, one study was published before 2010 [6] and fifteen studies were published between 2010 and 2020 [9–23].

**Table 1:** Characteristics of the studies included

References	Country	Study design	Sample size	Objective of the study	Scale	Statistical model	Effect size
1. Rwegerera et al. [21]	Botswana	Cross-sectional	380	To determine the HRQOL of DM patients in Botswana	SF-36	Logistic regression	OR & 95%CI
2. Tusa et al. [22]	Ethiopia	Cross-sectional	359	To assesses the level of HRQoL and its associated factors among adults with and without diabetes	WHO-QoL-BREF	Generalized Structural Equation Model	β and 95%CI
3. Gebremedhin et al. [14]	Ethiopia	Cross-sectional	267	To assesses the level of HRQoL and its associated factors among adults with and without diabetes	WHO-QoL-BREF	Linear regression	β and 95%CI
4. Reba et al. [20]	Ethiopia	Cross-sectional	344	To assesses the level of HRQoL and its associated factors among adults with and without diabetes	WHO-QoL-BREF	Linear regression	β and 95%CI
5. Aschalew et al. [10]	Ethiopia	Cross-sectional	408	To assess the HRQOL & associated factors of diabetic patients at the University of Gondar referral hospital, Ethiopia	WHO-QoL-BREF	Linear regression	β and 95%CI
6. Muze et al. [18]	Ethiopia	Cross-sectional	356	To assess quality of life and associated factors among diabetic patients having follow-up in diabetes clinic in Jimma University Specialized Hospital, Jimma, south west Ethiopia	SF-36	Logistic regression	OR & 95%CI
7. Ababio et al. [9]	Ghana & Nigeria	Cross-sectional	401	To assess QoL of patients with diabetes and to identify the predictors of good QoL among the patients with DM in the leading tertiary hospitals in Ghana and Nigeria	WHO-QoL-BREF	Linear regression	β and 95%CI
8. Jannoo et al. [16]	Mauritius	Cross-sectional	497	To formulate a hypothetical structural equation model linking HRQoL, diabetes distress, diabetes self-care activities, medication adherence and diabetes-dependent QoL in patients with Type 2 DM	SF-6 and ADDQoL-19	Structural Equation Model	β and 95%CI
9. Ekwunife et al. [13]	Nigeria	Cross-sectional	226	To assess the sensitivity of the EQ-5D instrument in a sample of Nigerian patients with type 2 diabetes mellitus (T2DM)	EQ-5D	Linear regression	β and 95%CI
10. Bolarinwa et al. [11]	Nigeria	Cross-sectional	59	To assessed the pattern and predictive factors of HRQoL among patients with hypertension, diabetes and concomitant hypertension and diabetes using the 36-item short-form version 2	SF-36	Linear regression	β and 95%CI
11. Odili et al. [6]	Nigeria	Cross-sectional	112	To assess the impact of diabetes on the health-related quality of life of Nigerians	WHO-QoL-BREF	-	-
12. James et al. [15]	Nigeria	Cross-sectional	212	To determine the relationship between depression and the subjective assessment of quality of life (QoL) in a sample of patients with diabetes mellitus (DM) attending outpatient clinics at a regional university teaching hospital in Nigeria	WHO-QoL-BREF	Linear regression	β and 95%CI
13. Werfalli et al. [23]	South African	Cross-sectional	341	To examine the prevalence of self-report diabetes, and association between diabetes and each of health-related quality of life and disability amongst South Africa’s older adults	WHO-QoL-BREF	Linear regression	β and 95%CI
14. Daya et al. [12]	South African	Cross-sectional	200	To determine the HRQOL of a sample of patients with type 2 diabetes	D-39	-	-
15. Mngomezulu et al. [17]	Swaziland	Cross-sectional	340	To understand the QOL and its correlates in diabetic outpatients in Swaziland	D-39	Linear regression	β and 95%CI
16. Nyanzi et al. [19]	Uganda	Cross-sectional	219	To assessing the factors associated with quality of life among diabetic patients in Uganda	QOLID	Poisson regression	Ratio rate

Regarding the tools that used to assess the level of HRQoL, Eight studies were used WHO-QoL-BREF [6, 9, 10, 14, 15, 20, 22, 23], three studies were used short-form-36 (SF-36) [11, 18, 21], two studies were used D-39 [12, 17], each one study was used EQ-5D [13], QOLID [19] and both SF 36 and ADDQoL-19 [16].

In our selected studies, eight studies were applied linear regression so their effect size was β with 95% CI [9–11, 13–15, 17, 20, 23], two studies were applied structural equation model so their effect size was β with 95%CI [16, 22], two studies were applied logistic regression so their effect size was OR with 95%CI [18, 21] and one study was applied a Poisson regression so their effect size was ratio rate[19].

### Quality of studies

JBI quality appraisal criteria established for analytical cross-sectional studies were used. The studies included in this systematic review and meta-analysis had no considerable risk. Therefore, all the studies were considered [6, 9–23] (Table 2).

**Table 2:** Quality of studies using Joanna Briggs Institute (JBI) criteria

References	Criteria for inclusion in the sample clearly defined	Study subjects and the setting described in detail	Exposure measured in a valid and reliable way	Objective, standard criteria used for measurement of the condition	Confounding factors identified	Strategies to deal with confounding factors stated	Outcomes measured in a valid & reliable way	Appropriate statistical analysis used	Scores	Overall quality
1. Rwegerera et al. [21]	Yes	Yes	Yes	Yes	No	Yes	Yes	Yes	7	Low risk
2. Tusa et al. [22]	Yes	Yes	Yes	Yes	No	Yes	Yes	Yes	7	Low risk
3. Gebremedhin et al. [14]	Yes	Yes	Yes	Yes	No	Yes	Yes	Yes	7	Low risk
4. Reba et al. [20]	Yes	Yes	Yes	Yes	No	Yes	Yes	Yes	7	Low risk
5. Aschalew et al. [10]	Yes	Yes	Yes	Yes	No	Yes	Yes	Yes	7	Low risk
6. Muze et al. [18]	Yes	Yes	Yes	Yes	No	Yes	Yes	Yes	7	Low risk
7. Ababio et al. [9]	Yes	Yes	Yes	Yes	No	Yes	Yes	Yes	7	Low risk
8. Jannoo et al. [16]	Yes	Yes	Yes	Yes	No	Yes	Yes	Yes	7	Low risk
9. Ekwunife et al. [13]	Yes	Yes	Yes	Yes	No	Yes	Yes	Yes	7	Low risk
10. Bolarinwa et al. [11]	Yes	Yes	Yes	Yes	No	Yes	Yes	Yes	7	Low risk
11. Odili et al. [6]	Yes	Yes	Yes	Yes	No	No	Yes	No	5	Low risk
12. James et al. [15]	Yes	Yes	Yes	Yes	No	Yes	Yes	Yes	7	Low risk
13. Werfalli et al. [23]	Yes	Yes	Yes	Yes	No	Yes	Yes	Yes	7	Low risk
14. Daya et al. [12]	Yes	Yes	Yes	Yes	No	No	Yes	No	5	Low risk
15. Mngomezulu et al. [17]	Yes	Yes	Yes	Yes	No	Yes	Yes	Yes	7	Low risk
16. Nyanzi et al. [19]	Yes	Yes	Yes	Yes	No	Yes	Yes	Yes	7	Low risk

### Meta-analysis

In our 16 selected studies the following different scales were used to measure level of HRQoL among diabetic patients; WHO-QoL-BREF (consisting of four domains: physical health, psychological health, social relation, and environmental health), SF-36 (consists of 8 domains (physical functioning, role physical, bodily pain, general health, vitality, social functioning, role emotional and mental health) and two composite scores (Physical Composite score & Mental Composite score)), D-39 (consists of 5 domains: diabetes control, anxiety and worry, social burden, sexual functioning, energy and mobility), EQ-5D (consists of 5 domains: Modality, Self-Care, Usual Activities, Pain/Discomfort and Anxiety/Depression), QOLID (consists of 5 domains: role limitation, mental health, treatment satisfaction, physical endurance, and diet satisfaction) and ADDQoL-19 (consists 19 items question). As each measure of scales have different domains, their result could not pooled by methods of meta-analysis. Therefore, we made a separate meta-analysis by their scale of measurement.

### Pooled level of HRQoL using domain of WHO-QoL-BREF

Among 16 selected studies, eight of them were used WHO-QoL-BREF [6, 9, 10, 14, 15, 20, 22, 23] to measure levels of HRQoL among diabetes patients. From these eight studies, five of them have reported the mean of each domain HRQoL. The summary of the Pooled mean for each domain of WHO-QoL-BREF and their associated factors, along with I-square and Egger’s regression test p-value is shown in Table 3.

**Table 3:** Summary results of Meta-analysis for each domain of WHO-QoL-BREF

Pooled mean/factors	Domains of WHO-QoL-BREF	Pooled mean/β with 95% CI	Egger's test	I-square (%)	Model
Pooled mean	Physical health	43.12 (29.10, 57.28)	0.048	99.8%	Random effects model
	Psychological health	47.40 (30.54, 64.25)	0.190	99.9%	Random effects model
	Social relation	46.60 (19.48, 73.71)	0.005	99.9%	Random effects model
	Environmental health	45.59 (36.31, 54.87)	0.083	99.7%	Random effects model
Age	Physical health	− 0.47(− 0.66,− 0.29)	–	0.01%	Fixed effects model
	Psychological health	− 0.24 (− 0.36, − 0.13)	–	76.9%	Random effects model
	Social relation	− 0.32 (− 0.44, − 0.20)	–	87.8%	Random effects model
	Environmental health	− 0.03 (− 0.04, − 0.01)	–	78.6%	Random effects model
Residence					
Urban	Environmental health	Ref	–	Ref	Fixed effects model
Rural		− 3.31 (− 5.15,− 1.47)	–	0.01%	
FBS	Physical health	− 0.08 (− 0.11, − 0.05)	–	0.01%	Fixed effects model
	Psychological health	− 0.08 (− 0.128, − 0.032)	–	68.8%	Random effects model
	Environmental health	− 0.004 (− 0.006,− 0.002)	–	87.9%	Random effects model
DRC					
No	Physical health	Ref	–	Ref	Fixed effects model
Yes		− 6.25(− 8.32, − 4.18)	–	45.6%	
Co-morbidity					
No	Physical heath	Ref	–	Ref	Fixed effects model
Yes		− 5.65 (− 7.90, − 3.40)	–	0.01%	

DRC, diabetic related complication; FBS, fasting blood sugar; Ref, reference

The pooled mean score of physical health, psychological, social relation and environmental health are 43.12 (95% CI; 29.10, 57.28), 47.40 (95% CI; 30.54, 64.25), 46.60 (95% CI; 19.48, 73.71) and 45.59 (95% CI; 36.31, 54.87) respectively. For each domain of WHO-QoL-BREF, I-square results confirm the presence of heterogeneity and for that we applied random model analysis. Egger’s regression test p-value is greater than 0.05 for psychological and environmental health domain of WHO-QoL-BREF, which indicated the absence of publication bias.

### Factors associated with level of HRQoL using domain of WHO-QoL-BREF

From eight studies that used WHO-QoL-BREF [6, 9, 10, 14, 15, 20, 22, 23], three of them had reported factors associated each domain of WHO-QoL-BREF. Age [10, 14], occupational status[10], exercise [10], general diet [10], diabetic self-care activity [22], medication adherence[22], Duration of disease [14], fasting blood sugar level [14, 22], and diabetes related complications [10, 22], number of complication[14], comorbidity[10, 14] and depression [22] were involved factors associated with physical health domain of WHO-QoL-BREF.

Age [10, 14], residence [22], marital status [10], exercise [10], general diet [10], sensible drinker [10], body mass index (BMI) [14], duration of disease [14], fasting blood sugar level [14, 22], diabetic related complication [22], number of complication [14] and depression [22] were included variables related with psychological health domains of WHO-QoL-BREF. Age [10, 14], residence [22], marital status [10], BMI) [14], diabetic self-care activity [22], duration of disease [14], fasting blood sugar level [14], diabetes related complications [10], number of complication [14] and depression [22] were significant variable associated with social relation domain of WHO-QoL-BREF.

Age [14, 22], residence [10, 22], marital status [22], educational status [10], foot care [10], general diet [10], body mass index [14], diabetic self-care activity [22], medication adherence [22], duration of disease [14], fasting blood sugar level [14, 22], diabetes related complication [10] and depression [22] were included factors related to the environmental health domain of WHO-QoL-BREF.

Age had a significant association (pooled β = − 0.47, 95% CI − 0.66, − 0.29), (pooled β = − 0.24, 95% CI − 0.36, − 0.13), (pooled β = − 0.32, 95% CI − 0.44, − 0.20) and (pooled β = − 0.03, 95% CI − 0.04, − 0.01) with physical health, psychological health, social relation and environmental health domains respectively. Being rural residence (pooled β = − 0.32, 95% CI − 0.44, − 0.20) was inversely associated with environmental health domain of WHO-QoL-BREF.

Fasting blood sugar level, Co-morbidity and diabetic related complication were important clinical factors that have a significant association with the domain of WHO-QoL-BREF. Increased fasting blood sugar had a significant association (pooled β = − 0.08, 95% CI − 0.11, − 0.05), (pooled β = − 0.08, 95% CI − 0.128, − 0.032) and (pooled β = − 0.004, 95% CI − 0.006, − 0.002) with physical health, psychological health and environmental health domains respectively. Having Co-morbidity (pooled β = − 6.25, 95% CI − 8.32, − 4.18) and diabetes related complication (pooled β = − 5.65, 95% CI − 7.90, − 3.40) were contrarily related with physical health domain of WHO-QoL-BREF.

**Fig. 3:**
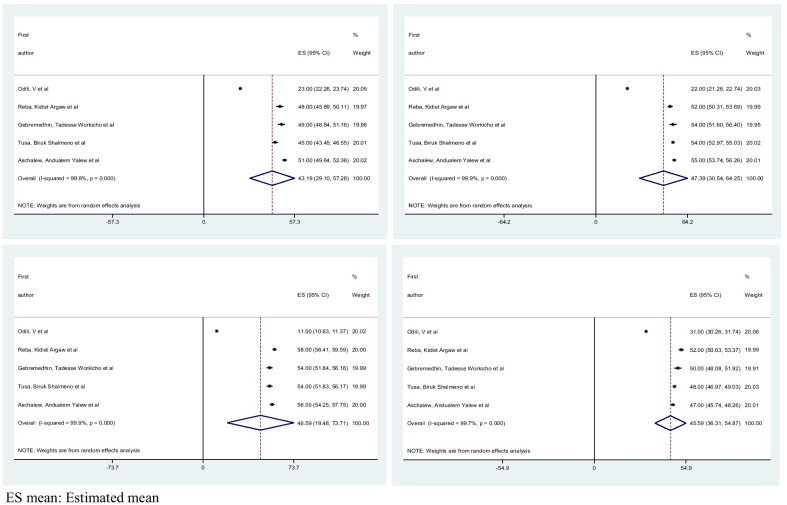
Forest plot of the pooled mean for each domain of WHO-QoL-BREF with corresponding 95% CIs

**Fig. 4:**
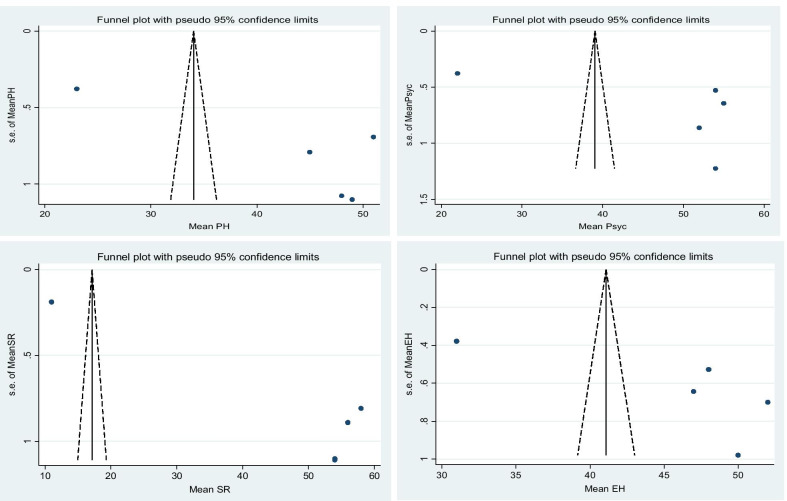
A funnel plots for publication bias for each domain of WHO-QoL-BREF

Figures 3 and 4 show forest plot of the pooled mean for each domain of WHO-QoL-BREF with corresponding 95% CIs and a funnel plot for publication bias for each domain of WHO-QoL-BREF respectively.

### Pooled level of HRQoL using short form-36

From 16 chosen studies, three of them were utilized short form-36 [11, 18, 21] to measure level of HRQoL among diabetic patients. Among these three studies, one study [18] had reported only the mean of each domain, one study [21] had reported only the mean of each composite scores and one study [11] had reported both the mean of each domain and composite scores.

**Fig. 5:**
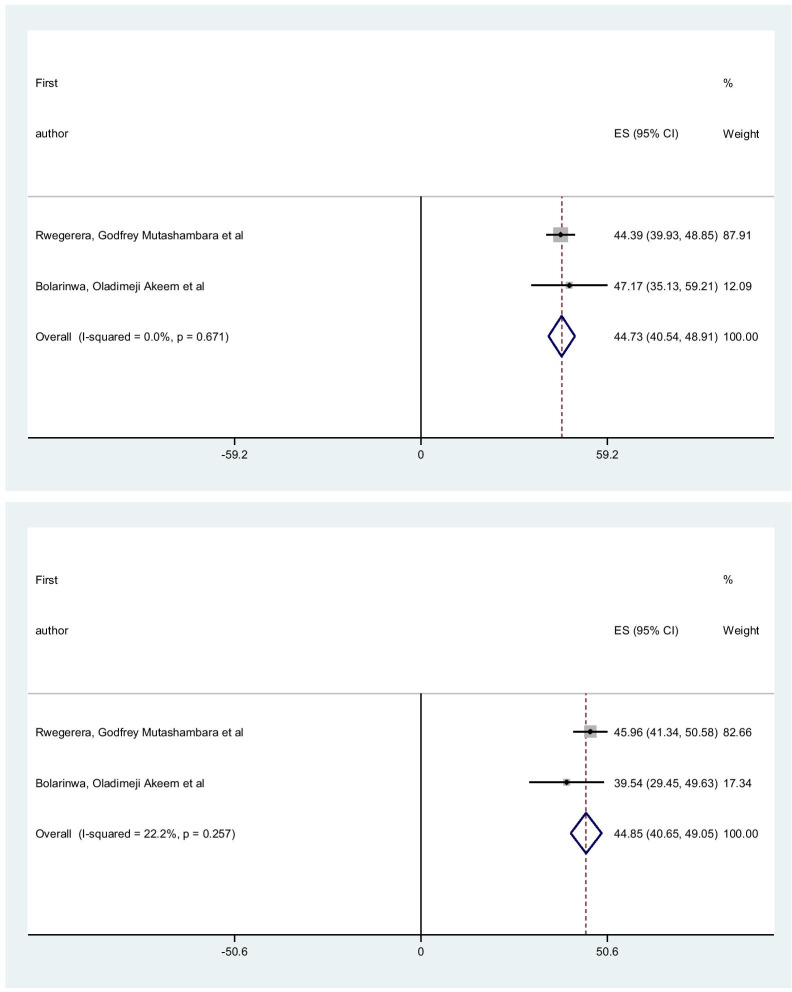
Forest plot of the pooled mean for each composite score of short form-36 with corresponding 95% CIs

The pooled mean of the physical composite score (PCS) and mental composite score (MCS) are 44.73 (95% CI 40.54, 48.91) and 44.85 (95% CI 40.65, 49.05) respectively. I-square for PSC & MSC were 0.01% and 22.2% respectively. This result indicated that the absence of heterogeneity (Fig. 5).

### Factors associated with level of HRQoL using domain of short form-36

Among three studies that use short form-36, two studied had presented factors associated with each composite score of short form-36, while one study had presented factors associated with each domain of short form-36.

Age [21], gender [21], diabetes related complication [21], drug combination [11], cardiovascular disease complication [11], accompanying person [11] are factors that were related to physical composite score of short form 36. Whereas diabetic related complication [21], Muskulo-skeletal disease [21] and medication adherence [11] are variables that have a significant association with mental composite score of short form 36.

## Discussion

Health care providers can recognize the physiological imbalance and the degree of deteriorations due to diabetes through different laboratory investigation. However, to improve the performance of everyday life activities and HRQoL among diabetic patients, health care providers want to recognize the physical, emotional and social influence of having a chronic disease and such patient centered knowledge can integrate with the existing diabetic mellitus treatment.

Health related quality of life of patients with diabetic is an important factor for analysis of the effectiveness of medications and other care [5]. Assessing HRQoL is essential, because they forecast the individuals’ ability to cope with his disease and sustain long term health &well-being and it is also progressively recognized as an significant health outcome in its own right, representing the ultimate ends of all health intervention [31].

In the present systemic review and meta-analysis a total of sixteen studies have been used to summarize the pooled level HRQoL and their associated factors among diabetic patients in sub-Saharan countries. These selected studies contained eight countries with 4721 diabetic patients and used six different kinds of scales to measure the level of HRQoL.

According to this meta-analysis the physical health was the most affected domain of the WHOQOL-BREF. Similarly the physical composite score of short form 36 have lower result than mental composite score. The possible explanation for these results, diabetes mellitus have more physical symptoms than psychological symptoms [32]. This could also be pronounced by patients with DM, have chance to develop diabetic related complications such as diabetic foot, which can interrupt their physical capacity to do their daily activities [33].

The current meta-analysis identified that a number of factors that were significantly associated with HRQoL including age, place of residence, fasting blood sugar, diabetic related complication and co-morbidities.

The present meta-analysis documented that older age was inversely associated with all domains of HRQoL. Such result could show that younger people are more likely to adore the quality of life than elder and this may not amazing because as age rise the physiological.

function drop and inhibit diverse activity of the body which might impair HRQOL [22]. Moreover, as the aging process leads to a degeneration of muscles, ligaments, bones, and joints and this problem become more and more in diabetes patients [10].

Being rural residence was contrarily associated with environmental health domain of HRQoL. This might be diabetic patient who are rural dwellers might have low knowledge about diabetic mellitus and also may not have sufficient access to health facility or professional support to maintain good glycemic control. Furthermore, urban dwellers have more access to information through different media and living standards.

Fasting blood sugar level, Co-morbidity and diabetic related complication were the identified clinical factor through this meta-analysis. Increased fasting blood sugar was inversely related to all domain of HRQoL except social relation. This might be due to increased fasting blood sugar have manifestations like excessive urination, excessive thirsty, excessive hunger, general weakness and sleeping disturbances [34], which may impair HRQoL. This can also be defensible as those who have increased fasting blood sugar want more health care services to maintain good glycemic control, are powerless to accomplish their day to day activities and are hopeless to join in different activities, paying to impair HRQoL [22].

Presence diabetic related complication and Co-morbidity were inversely associated with physical health domain of HRQoL. As diabetes patients have developed diabetic related complication and co-morbidity, it increases the number of medication they took and more money is needed to afford these drugs, need consider the amount of time for treating and hospital admission. This might be also be due to the influences of diverse chronic diseases in patients with diabetes and the side effects/drug interactions of the several drugs [14, 22]. More specifically, those who develop diabetic foot ulcer have anxiety to potential of amputation [33] which might result impairing of HRQOL of DM patients.

This systematic review and meta-analysis was the territorial estimation conducted in sub-Saharan nations. It may be lacking regional representativeness since data were not found from numerous countries of Sub-Saharan Africa. The included studies have utilized numerous distinctive HRQoL scales and resulting highly heterogeneous, so numerous results in our study were pooled by only 2 included articles. Moreover, the current study did not consider the number and types of covariates from different studies, while calculating the pooled effect size (Beta coefficients). This might be biased the pooled effect size.

## Conclusion

The pooled mean of physical and environmental domains of HRQOL scores were the least compared to the psychological and social and domains. Being Old age, rural residence, increased fasting blood sugar, having co-morbidity and diabetic related complications were contrarily related level of HRQoL. Therefore, we recommend that early detection and treatment of diabetes related complication and comorbidity and control of fasting blood sugar. While doing that due attention should be given for old ages and rural dwellers.

## Data Availability

All necessary information’s and supplementary materials were included with in the manuscript.

## Abbreviations

BMI: Body Mass Index
CI: Confidence interval
DM: Diabetic mellitus
HRQoL: Health related quality of life
JBI: Joanna Briggs Institute
MCS: Mental composite score (MCS)
PCS: Physical composite score
PRISMA: Preferred Reporting Items for Systematic Review and Meta-Analysis statement
PROSPERO: Protocol for this review was registered on the International Prospective Register of Systematic Reviews
OR: Odd ratio
QoL: Quality of life
